# Magnesium: Potential Roles in Neurovascular Disease

**DOI:** 10.3389/fneur.2014.00052

**Published:** 2014-04-15

**Authors:** Jason J. Chang, William J. Mack, Jeffrey L. Saver, Nerses Sanossian

**Affiliations:** ^1^Department of Neurology, University of Southern California, Los Angeles, CA, USA; ^2^Department of Neurosurgery, University of Southern California, Los Angeles, CA, USA; ^3^The Roxanna Todd Hodges Comprehensive Stroke Clinic, University of Southern California, Los Angeles, CA, USA; ^4^Department of Neurology, UCLA Stroke Center, University of California Los Angeles, Los Angeles, CA, USA

**Keywords:** magnesium, neuroprotection, intracerebral hemorrhage, subarachnoid hemorrhage, all cerebrovascular disease/stroke

## Abstract

**Objective:** Magnesium therapy has been studied extensively in pre-clinical and clinical trials in multiple organ systems. Cerebrovascular diseases may benefit from its neuroprotective properties. This review summarizes current studies of magnesium in a wide range of neurovascular diseases.

**Methods:** We searched relevant terms in the National Library of Medicine PubMed database and selected research including basic science, translational reports, meta-analyses, and clinical studies.

**Results:** Studies examining magnesium administration in ischemic stroke have failed to show any benefit in clinical outcome. Data on magnesium for intracerebral hemorrhage (ICH) are limited. Preliminary investigations in subarachnoid hemorrhage (SAH) were promising, but definitive studies did not reveal differences in clinical outcome between magnesium and placebo-treated groups. Studies examining magnesium administration in global ischemia following cardiac arrest suggest a trend toward improved clinical outcome. The strongest evidence for clinically relevant neuroprotection following magnesium administration derives from studies of pre-term infants and patients undergoing cardiac bypass and carotid endarterectomy procedures. Magnesium was found to have an excellent safety profile across all investigations.

**Conclusion:** Magnesium is easy to administer and possesses a favorable safety profile. Its utility as a neuroprotectant in cardiac surgery, carotid endarterectomy, and pre-term infant hypoxia remain promising. Value as a therapeutic agent in ischemic stroke, ICH, and SAH is unclear and appears to be limited by late administration. Ongoing clinical trials assessing magnesium administration in the first hours following symptom onset may help clarify the role of magnesium therapy in these disease processes.

## Introduction

Cerebrovascular diseases are the fourth leading cause of death in the United States, claiming one life every 4 minutes and affecting 15 million people worldwide yearly (1, 2). The identification of safe, effective, inexpensive, and easily applicable treatments is of enormous public health importance. Cerebral ischemia is the most common neurovascular disease, followed by intracerebral hemorrhage (ICH) and subarachnoid hemorrhage (SAH). Although these disorders have distinct mechanisms of brain injury, the neurovascular unit is principally affected in each. Treatments targeting both neuronal and vascular mechanisms may have efficacy in wide-ranging processes.

Magnesium (Mg) exhibits beneficial effects through both neuronal and vascular mechanisms, rendering it an attractive therapeutic agent in cerebrovascular diseases. Mg, an essential element for life, is found in virtually every biological system. All cells require Mg as an essential ion that allows ATP to be biologically active in DNA and RNA synthesis. Unique among neuroprotective agents, Mg has shown potential efficacy in a wide variety of clinical settings.

This review aims to summarize the current state of clinical knowledge regarding Mg therapy in the setting of cerebrovascular diseases. We begin with a brief summary of pre-clinical data. However, extensive review of experimental results is beyond the scope of the article. We focus on reported clinical trials and meta-analyses, segregated according to disease process. We conclude with a discussion of a current clinical trial and future directions in research.

## Pre-Clinical Data for Magnesium

Mg exhibits multiple complementary neuroprotective mechanisms. Physiological extracellular Mg concentrations (250–1000 μmol/L) inhibit glutamate release (3), potentiate adenosine-mediated inhibition of glutamate release (4), restore blood–brain-barrier (BBB) integrity, decrease brain edema (5), and non-competitively antagonize NMDA receptor activation via blockage of voltage-dependent calcium channels (6). In rat brain injury models, intracellular Mg has been linked to alterations in cerebral energy metabolism and inhibition of mitochondrial function (7). Finally by competing with calcium at voltage-gated calcium channels in intracellular and cell surface membranes and by serving as an antagonist at N-type, P-type, and L-type channels, Mg impedes calcium influx into ischemic neurons and prevents a recognized final common pathway for cell death (8).

Mg possesses vasoactive properties, which can improve cerebral blood flow via interactions with the regional vasculature and more distant effects in the heart and systemic circulation. In the cerebral vasculature, Mg exhibits direct effects on large, medium, and small vessels by inhibiting the actions of endothelin-1, neuropeptide Y, and angiotensin II (9, 10). In rabbit models, inhibition of L-type smooth muscle Ca channels suggests a potential role in vasospasm prevention (11). Augmentation of cerebral blood flow via vasodilatation results in improved outcomes following experimental middle cerebral artery occlusion in animal models (12). Systemically, Mg promotes vasodilatation by increasing synthesis of prostacyclin and inhibition of angiotensin (13). Mg also augments cardiac output, increasing cardiac index despite a mild transient lowering of systemic blood pressure (14).

## Evaluation of Physiological Magnesium Concentration

Low serum Mg levels (<0.76 mmol/L), coupled with advanced atherosclerosis, are associated with a 3.29-fold increased adjusted risk of adverse cerebrovascular events (95% CI 1.34–7.90, *p* = 0.009) (15) and promote coronary and carotid atherosclerosis (16). These studies did not demonstrate a correlation between serum Mg levels and Glasgow Coma Scale. However, lower cerebrospinal fluid (CSF) Mg levels were present in patients with ischemic strokes compared to controls (*p* = 0.006) and established a positive association between mortality after 7 days and significantly lower CSF levels of Mg (*p* = 0.002) (17). During cardiac bypass surgery (CABG), intra-operative serum Mg levels were found to be low during an initial stage of the operation (18).

The ability of Mg to permeate intact BBB and enter CSF spaces has remained controversial. Initial studies on preeclampsia patients revealed that CSF concentrations were only modestly but consistently elevated in patients who received intravenous Mg therapy (19). Brain penetration of Mg is thought to be enhanced by BBB dysfunction (20). However, patients with chronic neurological diseases with presumed mild BBB disruption do not have significant differences in CSF Mg concentrations compared to healthy controls (21). In addition, patients with acute neurological injury and induced intravenous hypermagnesemia were shown to produce only modest increases in CSF levels of total and ionized Mg (22).

## Clinical Trials of Magnesium Sulfate in Neurovascular Disease

### Cerebral ischemia

Six phase 2 trials have examined Mg sulfate administration in ischemic stroke (23–28). Each of the phase 2 trials demonstrated safety with Mg, but did not demonstrate improved clinical outcome with Mg. A meta-analysis of four of the phase 2 randomized controlled trials with analogous data in 162 patients found a trend toward improved clinical outcome, with late disability or death seen in 44.3% of Mg sulfate patients compared with 52.7% of placebo patients (OR = 0.67, 95% CI = 0.35–1.26) (29). Furthermore, individual phase 2 trials optimized a dosing regimen to achieve target doubling of serum Mg levels and demonstrated feasibility of pre-hospital initiation of therapy (24, 27).

The largest study to date that examined Mg therapy in acute ischemic stroke was the phase 3 intravenous magnesium efficacy in stroke (IMAGES) trial (28). IMAGES enrolled 2589 patients within 12 h of symptom onset. Patients were randomized to either 20 g Mg sulfate (4 g bolus followed by 16 g maintenance given over 24 h) or matched placebo treatment. The primary endpoint as measured by death and disability at 3 months and identified by the joint binary outcome of Barthel score <95 and a modified Rankin score (mRS) >1, did not reach statistical significance (OR = 0.95, 95% CI = 0.80–1.13).

Intravenous magnesium efficacy in stroke had three critical shortcomings. First was late enrollment with median time to treatment of 7 h. Only 71 patients (3.1% of the entire IMAGES cohort) were enrolled within 3 h of onset and only 16 (0.6%) were enrolled in the first 2 h. Second, IMAGES required that a patient’s deficit be persistent for at least 1 h before enrollment could occur, precluding initiation of Mg within the first 60 min of stroke onset. Third, as Mg is an endogenous ion and Mg serum levels were not evaluated nor hypomagnesemia screened, it is unclear whether there was a difference in Mg levels between the Mg and control group.

And although aggregate results did not demonstrate improved clinical outcome with Mg, the trial suggested a potential benefit in patients who were administered treatment at earlier time points. Among patients enrolled in the first 3 h, favorable trends were noted: a non-disabled outcome (mRS 0–1) was achieved in 45.9% of Mg patients vs. 33.3% of placebo patients and death was observed in 26% of placebo vs. 19% of Mg patients (OR = 0.65, 95% CI = 0.23–1.92) (28). These trends allowed the possibility that a sufficiently powered trial utilizing earlier administration of Mg could provide evidence for potential efficacy of Mg in ischemic stroke.

### Intracerebral hemorrhage

The largest study to date examining Mg in ICH is IMAGES. Use of Mg prior to brain imaging resulted in 168 (8.3%) of the total enrolled patients being diagnosed as ICH. The point estimate of effect in reducing death or disability was favorable (OR = 0.84, 95% CI = 0.41–1.74) in this cohort, although the sample size was too small to draw definitive conclusions (28). Of particular relevance to ICH are the vasodilatory and antihypertensive effects of Mg. At the dose tested in IMAGES and FAST-MAG, Mg can lower systolic blood pressure by 3–4 mm Hg. Although failing to meet its primary outcome, INTERACT2 did show a trend suggesting decreased death and major disability with intensive blood control (*p* = 0.06) and did show significantly lower mRS via ordinal analysis (*p* = 0.04) (30). Whether Mg can improve clinical outcome in ICH through blood pressure control or neuroprotection remains to be seen.

In the FAST-MAG Phase 3 trial, initial evaluation of the first 750 enrolled patients yielded 24% of patients with a final diagnosis of ICH, which anticipates a subgroup of approximately 400 patients to further explore potential benefit of Mg in ICH.

### Delayed cerebral infarction after subarachnoid hemorrhage

Hypomagnesemia occurs in about 50% of SAH patients and is associated with a high risk of delayed cerebral ischemia and poor 3-month outcome. The promise of Mg as both a neuroprotective and vasodilatory agent led to clinical pilot trials in aneurysmal SAH where Mg administration was associated with reductions in symptomatic vasospasm and favorable trends in clinical outcome (31, 32). These pilot trials led to the magnesium and acetylsalicylic acid in subarachnoid hemorrhage (MASH-1) trial, a European phase 2b trial that randomized 283 patients with aneurysmal SAH to Mg sulfate (64 mmol/L/day) or placebo. Mg therapy tended to reduce the incidence of delayed cerebral infarction on 3-month head CT by 34% (HR = 0.66, 95% CI = 0.38–1.14) and the 3-month risk of death or disability by 23% (OR = 0.77, 95% CI = 0.54–1.09). However, only improvement in excellent outcome as assessed by Rankin scores of 0 was significant (33).

Unfortunately, subsequent phase 3 SAH trials have not been confirmatory. In IMASH (Hong Kong Coordinating Center), 327 patients with aneurysmal SAH within 48 h of onset were randomized to serum Mg concentrations twice that of baseline or placebo therapy for 10–14 days. Serum Mg levels were significantly higher in the Mg group (1.67 mmol/L) than the placebo group (0.91 mmol/L) (*p* < 0.001). Primary 6-month favorable outcome rates – measured by an extended Glasgow Outcome Scale score of 5–8 – in the Mg (64%) and placebo group (63%) were similar (OR = 1.0, 95% CI = 0.7–1.6). Secondary outcome measures – Rankin scores (<2), Barthel Index (>85), clinical vasospasm, decrease in Glasgow Coma Scale (≥2), and relevant complications – were similar between the two groups (34).

The subsequent phase 3 MASH-2 trial (1204 patients, Netherlands Coordinating Center) also revealed no difference between patients treated with 64 mmol/day of Mg sulfate or placebo therapy. Inclusion criteria required aneurysm presence and participation within 4 days of onset. Duration of Mg treatment was 20 days post SAH-onset or until hospital discharge. Primary outcome was dichotomized. Poor outcome, defined as 3-month Rankin scores of 4–6, was similar for the Mg (26.2%) and placebo groups (25.3%) (RR = 1.03, 95% CI = 0.85–1.25). Distribution of Rankin scores was not statistically different between the two groups. Subgroup analysis did not identify specific subgroups that might have benefited from Mg (35).

The time window for Mg administration may have limited the potential efficacy of Mg in these trials. The mean time to initiation of therapy was 31.7 h in IMASH and 33 h in MASH-2. However, the processes leading to delayed cerebral ischemia after vasospasm may be present by that time, and this latency may not have been detrimental. Another possible contributor to lack of efficacy may be BBB penetration. Large, acute ischemic strokes typically open the BBB over substantial regions, allowing CSF penetration of Mg. However, the vasospasm associated with SAH is of subacute onset and may be associated with less disruption of the BBB at the time of ischemia. Therefore, Mg may not be able to effectively cross into the CSF spaces at relevant time points. Further complicating the analyses is that serum Mg levels were not measured as study parameters in MASH-2.

### Global cerebral ischemia after cardiac arrest

Pre-hospital administration of magnesium sulfate showed favorable trends toward neuroprotection in resuscitated cardiac arrest patients in the brain-cardiopulmonary resuscitation (B-CPR) trial. The B-CPR trial tested Mg sulfate and diazepam in a 2 × 2 factorial, placebo-controlled design. Paramedic personnel randomized subjects and administered study medications to out-of-hospital cardiac arrest patients immediately following return of spontaneous circulation. Three hundred patients were randomized to intravenous Mg sulfate (2 g) or placebo, and then to diazepam (10 mg) or placebo. Treatment was administered in the field by paramedics but was not continued during hospitalization. Patients receiving Mg demonstrated significantly higher serum Mg levels (3.0 mg/dL in the Mg group vs. 2.1 mg/dL in the placebo group).

The pre-specified primary outcome measure of awakening at any time by 3 months after cardiac arrest was seen in 46.7% of patients treated with Mg and 37.3% of those treated with placebo (risk difference 9.3%, CI = 6.4–25.1%). The diazepam cohort showed no evidence of treatment effect. No adverse effects of pre-hospital administration of Mg sulfate therapy were noted. Limitations of this study included failure of randomization to produce balanced treatment groups and uncertainty as to whether continuation of treatments may have altered final outcomes (36).

### Pre-term infant hypoxic–ischemic injury and intraventricular hemorrhage

Pre-term infants are at high risk of hypoxic–ischemic perinatal brain injury and resultant cerebral palsy or death. Although a small, early trial (MAGnet) suggested increased mortality (37) and other trials raised concerns for possible lenticulostriate vasculopathy and intraventricular hemorrhage (38), subsequent larger trials have allayed these concerns and indicated a neuroprotective benefit of Mg in averting fetal brain injury in pre-term deliveries. Meta-analyses have demonstrated reduced mortality in the cohort of infants that received Mg (RR = 0.73, 95% CI = 0.61–0.89) (39) and shown that antenatal Mg given to women at risk of pre-term birth substantially reduced the risk of cerebral palsy in their child (RR = 0.69, 95% CI = 0.54–0.87) and gross motor dysfunction (RR = 0.61, 95% CI = 0.44–0.85) (40).

Three large randomized controlled trials have investigated the neuroprotective effects of Mg on the fetal brain when administered to mothers within 24 h of pre-term delivery. In ACTOMgS04 Trial, evaluation at 2 years showed lower frequency of gross motor dysfunction (3.4% Mg vs. 6.6% placebo; RR = 0.51, 95% CI = 0.29–0.91) and combined death or substantial gross motor dysfunction (17.0% Mg vs. 22.7% placebo; RR = 0.75, 95% CI = 0.59–0.96) in the Mg cohort (41). In the PREMAG Trial, infants receiving Mg demonstrated trends toward a decreased frequency of severe white matter injury (10.0% Mg vs. 11.7% placebo; OR = 0.78, 95% CI = 0.47–1.31) and total mortality (9.4% Mg vs. 10.4% placebo; OR = 0.79, 95% CI = 0.44–1.44) (42). Finally, a multicenter, placebo-controlled, double-blind trial showed that infants treated with Mg had significantly less frequent moderate or severe cerebral palsy (1.9% Mg vs. 3.5% placebo; RR = 0.55, 95% CI = 0.32–0.95), suggesting a potential neuroprotective role for Mg in pre-term infants who survive (43).

Mg has also been promising for prevention of intraventricular hemorrhage in pre-term infants. In a prospective study of 125 patients, 4 g of Mg sulfate and aminophylline was added to corticosteroids and ritodrine to treat newborns born before 30 weeks. This group was compared to newborns only receiving the standard regimen of corticosteroids and ritodrine. The rate of intraventricular hemorrhage in the Mg and aminophylline group was significantly lower (5.1%) than that of the control group (20.6%) (*p* < 0.001) (44).

The greater capacity for BBB penetration in pre-term infants may account for the substantial differences in neuroprotective efficacy between infants and adults treated with Mg sulfate therapy. However, Mg toxicity must be monitored in pre-term infants, who may be more vulnerable than adults owing to underdeveloped renal function and greater BBB penetration.

### Cardiac bypass surgery and intra-operative brain ischemia

Neuropsychological dysfunction is evident in up to 50–80% of CABG patients at the time of hospital discharge. Hypomagnesemia may result in cardiac tachyarrhythmias such as ventricular fibrillation, which may contribute to intra-operative hypoperfusion and subsequent neurological deficits. The Cleveland Clinic trial randomized 350 patients undergoing elective CABG to intra-operative Mg sulfate or placebo treatment that was continued for the first 24 post-operative hours. Patients received a 2-g loading dose and subsequent titration of a continuous infusion to maintain serum Mg levels at twice normal. The Mg cohort demonstrated significantly lower frequencies of neurologic decline and neurologic death at 96 h after surgery, when compared to the placebo-treated group (*p* = 0.01). At 3 months post-operatively, there were no performance differences between groups. This long-term data may reflect a test ceiling effect, as placebo patients appeared to improve over time (45).

### Carotid endarterectomy and neuroprotection

A preliminary prospective dose escalation study in 80 patients undergoing elective carotid endarterectomy established that Mg and placebo groups had statistically significant different serum levels of Mg (*p* < 0.01) and no significant difference in adverse events (*p* = 0.66) (46). The neuroprotective effect of Mg in carotid endarterectomy was evaluated in a randomized, double-blinded, placebo-controlled study of 108 patients. Patients undergoing carotid endarterectomy were divided into three stratified levels of Mg dosing (total intra-operative infusions of 10, 18, and 20 g) or placebo treatment. Another 35 patients undergoing lumbar laminectomy served as controls. Overall, patients treated with Mg had less post-operative neurocognitive impairment than those administered placebo (OR = 0.27, 95% CI = 0.10–0.74, *p* = 0.01). However, stratification by Mg levels did not affect outcomes in a dose-dependant manner. In fact, the low-dose Mg groups (10 and 18 g) demonstrated the greatest improvement (OR = 0.09, 95% CI = 0.02–0.50, *p* = 0.01), whereas the high-dose Mg group (20 g) showed no difference when compared to the placebo-treated cohort (47).

## Future Directions for Magnesium in Cerebrovascular Disease

As outlined in Table 1, magnesium therapy has demonstrated evidence of clinically relevant of clinically relevant neuroprotection in neurovascular disorders and procedures including global cerebral ischemia, pre-term neonatal hypoxia, carotid endarterectomy, and CABG. To date, however, pre-clinical promise has not translated to effective therapy in acute ischemic stroke, ICH, and SAH.

**Table 1:** **Summary of important clinical trials**.

	Vascular disease state evaluated	Sample size	Time administered and dosage	Outcome measure	Result	Conclusion	Key limitations
**IMAGES**							
Lees et al. (26)	1. Acute stroke (92% cerebral ischemia)	2589 (total)	Median 7 h 4 g bolus + 16 g infusion	Death or disability at 90 days (Rankin and Barthel score)	1. Ischemic stroke: death or disability not improved by Mg (OR = 0.95, 95% CI = 0.80–1.13, *p* = 0.59)	1. Ischemic stroke: late administration of Mg does not reduce death or disability	Long interval until Mg administration
	2. Intracerebral hemorrhage	168 (ICH)			2. ICH: death or disability non-significantly improved by Mg (OR = 0.84, 95% CI = 0.41–1.74)	2. ICH: Mg shows potential signal of efficacy in reducing death or disability	
**IMASH**							
Wong et al. (34)	Delayed ischemia after subarachnoid hemorrhage	327	Mean 31.7 h 80 mmol qday (×14 days)	Favorable score on GOSE (5–9) at 6 months	Favorable outcome not improved by Mg (OR = 1.0, 95% CI = 0.7–1.6)	Mg does not improve favorable outcome in subarachnoid hemorrhage	Long interval until Mg administration
**MASH-2**							
Dorhout Mees et al. (35)	Delayed ischemia after subarachnoid hemorrhage	1204	Median 33 h 64 mmol qday (×20 days)	Death or dependence (Rankin score 4–6) at 3 months	Dependence or death not improved by Mg (RR = 1.03, 95% CI = 0.85–1.25)	Mg does not improve favorable outcome in subarachnoid hemorrhage	1. Mg concentrations not routinely checked
							2. Long interval until Mg administration
**B-CPR TRIAL**							
Longstreth et al. (36)	Global brain ischemia after cardiac arrest	300	Pre-hospital 2 g bolus	Awakening by 3 months	Awakening non-significantly improved by Mg (adjusted HR = 1.14, 95% CI = 0.67–1.94)	Mg shows potential signal of efficacy in improving awakening	1. Randomization did not produce balanced treatment groups
							2. Initial treatments given by EMS, but not continued
**ACTOMgSO4**							
Crowther et al. (41)	Pre-term hypoxic–ischemic encephalopathy	1047	Expectant mothers (30 weeks) with pre-term labor 4 g bolus + 1 g/h infusion	Total mortality (≤2 year), cerebral palsy (≤2 year), or combined death + cerebral palsy (≤2 year)	1. Primary outcome improved by Mg (RR = 0.83, 95% CI = 0.66–1.03, *p* = 0.09)	1. Mg showed trend of lowering death or cerebral palsy	1. Lack of uniformity with cerebral palsy diagnosis
					2. Substantial decrease in motor dysfunction (RR = 0.51, 95% CI = 0.29–0.91, *p* = 0.02)	2. Mg significantly reduced substantial motor dysfunction	2. Neurosensory deficit did not necessarily correlate with clinical disability
							3. Long-term benefits of Mg unknown
**PREMAG**							
Marret et al. (42)	Pre-term hypoxic–ischemic encephalopathy	573	Expectant mothers (<33 weeks) with pre-term labor 4 g bolus	Mortality before hospital discharge	Mortality not improved by Mg (adjusted OR = 0.79, 95% CI = 0.44–1.44)	Mg does not increase pre-term infant mortality	1. No maintenance infusion of Mg used
							2. Study aborted early due to poor enrollment rate
Rouse et al. (43)	Pre-term hypoxic–ischemic encephalopathy	2241	Expectant mothers (24–31 weeks) with pre-term labor 6 g bolus + 2 g/h infusion (×12 h)	Mortality (≤1 year) and moderate/severe cerebral palsy at 2 years	1. Death or mod/severe cerebral palsy not improved by Mg (RR = 0.97, 95% CI = 0.77–1.23) 2. Risk of mod/severe cerebral palsy was improved by Mg (RR = 0.55, 95% CI = 0.32–0.95)	1. Mg did not improve mortality and development of cerebral palsy in pre-term infants 2. Mg significantly lowered moderate to severe cerebral palsy in pre-term infants	Composite primary outcome (death and cerebral palsy) chosen because death would lower prevalence of cerebral palsy; in the trial death was 3–4× more common than mod/severe cerebral palsy
Bhudia et al. (45)	Intraoperative ischemia in cardiac bypass	350	Perioperative 780 mg bolus + 3.16 g infusion	Post-op mortality, neurologic assessment^a^ at 24 and 96 h, neuropsychologic and depression assessment^b^ at 3 months	1. Mg decreased risk of neurologic decline (*p* = 0.0001) 2. Mg did not improve neuropsych performance at 3 months (*p* = 0.6)	Mg decreased neurologic decline post-op	Only small fraction of cardiac patients included due to exclusion factors
Mack et al. (47)	Intraoperative ischemia in carotid endarterectomy	108	Perioperative/stratified with low-dose receiving 2 g bolus + 8 g infusion	Neuropsychologic outcome at post-op day 1^c^	Mg decreased chance of neurocognitive impairment in low-dose Mg group only (OR = 0.09, 95% CI = 0.02–0.50, *p* < 0.01)	Mg decreased neurocognitive decline	Mg dose effect not observed

*^a^Neurologic assessment via Western Perioperative Neurologic Scale (WPNS)*.

*^b^Neuropsychologic and depression assessment consisting of Hopkins Verbal Learning Test, Controlled Oral Word Association Test, Boston Naming Test, and Digit Symbol and Symbol Search subtests of Wechsler Adult Intelligence Scale*.

*^c^Neuropsychologic outcome via neuropsychometric tests consisting of Boston Naming Test, Halstead–Reitan Trails Parts A and B, Controlled Oral Word Association Test, Copy Portion of Rey Complex Figure Test*.

Over the past several decades, more than 70 neuroprotective agents have been tested in randomized controlled clinical trials in acute ischemic stroke (48). Several agents, including Mg, have shown promising preliminary results followed by disappointing outcomes in definitive phase III trials. No neuroprotective therapy has been approved by the FDA for an indication of ischemic stroke. Leading neuroscientists have identified four key design failures in prior human clinical trials of neuroprotectants: (1) failure to select patients who will respond to the mechanisms of action of the study drug, (2) failure to administer study agents at neuroprotective doses in humans, (3) failure to employ sample sizes large enough to detect modest benefits of a study agent, and (4) failure to treat patients early enough after stroke onset (48–57). These four areas of design failure have now been addressed in more recent, sophisticated trials, starting with FAST-MAG. Primary results have yet to be published.

Future directions for Mg are likely to include combination neuroprotectant therapy. Mg may hold some benefit to ischemic stroke patients treated with reperfusion therapies, by preserving threatened brain tissue until restoration of blood flow. This hypothesis will be tested in sub-analyses of FAST-MAG where approximately 400 randomized patients will have received thrombolysis (58). In addition, studies have demonstrated an additive benefit when Mg is combined with hypothermia in rat ischemic stroke models (59–61). Currently, hypothermia combined with intravenous thrombolysis is being studied in a phase 3 clinical trial. Future treatment paradigms may include combination strategies with pre-hospital Mg administration and hypothermia followed by intravenous thrombolysis. The established safety profile of Mg may lead to an emerging role as a promising adjunct treatment. Sub-analyses from the completed FAST-MAG trial, coupled with the established body of research, will help determine Mg’s future role in cerebrovascular disease.

## Conflict of Interest Statement

The authors declare that the research was conducted in the absence of any commercial or financial relationships that could be construed as a potential conflict of interest.
